# Long Covid: A call for global action

**DOI:** 10.1002/puh2.69

**Published:** 2023-02-13

**Authors:** Arianna Maever Loreche, Veincent Christian F. Pepito, Manuel M. Dayrit

**Affiliations:** ^1^ Center for Research and Innovation, School of Medicine and Public Health Ateneo de Manila University Pasig Philippines; ^2^ National Clinical Trials and Translation Center, National Institutes of Health University of the Philippines Manila Manila Philippines

**Keywords:** COVID‐19, global health, Long Covid, pandemic, population health

## Abstract

The COVID‐19 pandemic has resulted in another global health crisis with millions of individuals with Long Covid experiencing disability, worse health‐related quality of life, and productivity and income losses. Despite its impact on population health outcomes, health systems, and the economy, there remains a need for a global consensus on its standard definition, diagnostic approaches, and treatment plans to manage symptoms. In many low‐ and middle‐income countries (LMICs), there are limited published estimates on Long Covid, limited studies on its diagnostics and therapeutics, and limited media and news coverage. We advocate for a global action plan and necessary investments on Long Covid research and treatment, meaningful involvement of patients and cooperation between high‐income countries and LMICs, stronger and more effective public health communication surrounding the risks of Long Covid, and support toward financial risk protection as advocated by Universal Health Care.

## BACKGROUND

There are more than 600 million reported COVID‐19 cases and more than 6 million deaths, with its true burden estimated to be much larger [1]. These two metrics have mainly been the focus of governments as they implemented disease control policies, including vaccination of populations. However, a significant number of individuals have reported prolonged symptoms and disability following COVID‐19 infection. In the early phase of the pandemic and primarily through social media, patients coined the term ‘Long Covid’ to characterize long‐lasting symptoms when infection was initially reported to be mild and recovery quick [2]. Since then, Long Covid has been integrated into formal health and policy channels [2] prompting studies on the burden of Long Covid, its pathophysiology, and development of appropriate diagnostic tests and effective treatment to address its impact.

At least 1 in 10 people experience persistent symptoms 12 weeks after having COVID‐19 [3], across an average of 9 organ systems affected [4, 5]. Hypotheses for its pathogenesis point to persisting reservoirs of SARS‐CoV‐2 in tissues, immune dysregulation with or without reactivation of underlying pathogens, autoimmunity, impacts on the microbiota, microvascular blood clotting, priming of the immune system from molecular mimicry, and dysfunctional signaling in the brainstem and/or vagus nerve. The National Center for Health Statistics estimates that the burden of Long Covid in the United States is around 15% [6], more than 2 million in the United Kingdom or 3.3% of the total population as reported by the Office for National Statistics [7], with the global prevalence estimated to be around 145 million after 3 months of infection [5, 8]. A global review of literature reported an estimated incidence of Long Covid at 10%–30% among nonhospitalized cases, 50%–70% among hospitalized patients, and 10%–12% among vaccinated cases. These figures mean that countries with high infection rates are being severely impacted by the burden of Long Covid, with negative consequences on the health system and economy.

A significant proportion of individuals with Long Covid experience productivity and income losses, worse health‐related quality of life, and an increased risk for suicidal ideation and behavior and mortality, among other negative health outcomes [9, 10, 11]. In the United States with the highest cumulative case count, more than a million people will be out of the workforce at any time period because of Long Covid with a projected USD 50 billion loss of income in a year [12]. Although Long Covid has been reported across all age groups and disease severities, the impact of Long Covid is even more pronounced among disadvantaged settings and marginalized groups including ethnic and racial minority groups [5], with a higher prevalence reported among individuals in lower income classes and among Hispanics and Latinos. Additionally, there is an increased risk for Long Covid among people with other health conditions, including type 2 diabetes, chronic urticaria and allergic rhinitis, connective tissue disorders, and those with prior illnesses such as infectious mononucleosis and mast cell involvement; however, a third have no identified health conditions.

Despite this mass disabling situation, governments have relaxed on public health measures because of perceptions that COVID‐19 is mild with a full recovery in a few weeks and optimism from world leaders [13]. There is also a lack of messaging on risks of developing Long Covid, with Facebook posts on Long Covid by US state health departments rare compared to COVID‐19 (Long Covid: 137 posts vs. COVID‐19: 49,310) [14]. Current vaccines only reduce the odds of getting Long Covid by around 8%–12% [15], and there remains a need for global consensus on its standard definition, diagnostic approaches, and treatment plans to manage the symptoms and long‐term sequelae.

## ADDRESSING LONG COVID: RESEARCH, DIAGNOSIS, AND TREATMENT

To end the COVID‐19 global health threat, a multidisciplinary panel of experts arrived at a series of consensus statements and recommendations that included prioritizing research funding for Long Covid and accelerating the development of effective therapies [16]. In 2021, the National Institutes of Health in the United States announced a USD 1.15 billion grant to support Long Covid research through the Researching COVID to Enhance Recovery (RECOVER) Initiative and the UK Government a GBP 18.5 million grant for four studies. These grants and other initiatives in high‐income countries (HICs), publicly funded or otherwise, have provided critical evidence on the determinants, burden, and outcomes of Long Covid.

In 2022, the United States published a national research action plan for Long Covid, with an emphasis on therapeutics and making mention of the NIH‐funded RECOVER Initiative [17]. Despite this action plan and a budget of nearly USD 470 million for clinical investigations, RECOVER has primarily been observational in nature and is anticipated to launch trials of treatments more than 12 months after the funding was announced [17, 18]. The prioritization of and progress on Long Covid are especially lagging in other regions, particularly low‐ and middle‐income countries (LMICs) with already fragile health systems and limited resources. Majority of published research are from HICs in the Americas and Europe, with underrepresentation in Africa and Asia [19, 20, 21]. From a recently published systematic review and meta‐analysis, only 30% of the 50 studies on the prevalence of Long Covid were from LMICs, including Bangladesh, Brazil, China, India, Iran, and Mexico [21]. In the Philippines, there are no published estimates on Long Covid, no published studies on diagnostics and therapeutics, and limited media and news coverage. These gaps in knowledge and dissemination in LMICs are in part not only due to the lack of funding but also because of limited capacities to diagnose and monitor Long Covid.

Compared to HICs, evidence on the long‐term effects of COVID‐19 in LMICs is limited [20]. In addition, children are an underrepresented population with data primarily reported from the United States [21]. Data reporting systems should be adaptive and flexible [22] to collect and analyze follow‐up data from individuals who test positive for the virus across all age groups. Such data repositories in HICs have enabled analyses such as modeling of phenotype data [23] and risks for cardiovascular disease and mortality events [24]. Additionally, available diagnostics and technologies in HICs have led to a better understanding of cellular and molecular mechanisms of Long Covid, which will facilitate the development of effective treatments. These include clinical biomarkers and screening technologies, which are being used in ongoing studies such as those in France (French COVID Cohort; ClinicalTrials.gov ID NCT04262921), Canada (MOIST Study; ClinicalTrials.gov ID NCT04525404), and the United Kingdom (PHOSP‐COVID) [25]. However, these are neither readily available nor affordable in LMICs.

Other options, therefore, need to be tested and explored, and collaborative work between HICs and LMICs is warranted [25]. In HICs, there have been efforts to develop guidance for Long Covid management with a panel of UK doctors generating a list of recommendations [26]. Patient involvement is critical to account for their lived experiences, and such guidelines will need to be regularly updated and adapted in other countries for tailored management.

## WAY FORWARD: GLOBAL ACTION PLAN AND COOPERATION

Given the burden of Long Covid in communities, governments, especially in LMICs, will need to allocate funds and resources for research, and patients and families inflicted with post‐acute sequelae of COVID‐19 similar to what the United States, United Kingdom, Germany, and other HICs have embarked on. Research and management modalities need to be patient‐centered and context‐specific. This will mean expanding the approach from biomedical and epidemiological to involve researchers from the social sciences. More importantly, there is a need for a unified research agenda to allow for robust and reproducible studies. This should include research on risk factors, diagnosis and ascertainment of Long Covid, long‐term effects of COVID‐19 such as interactions between COVID‐19 infection and other comorbidities, effective therapies and management, and lived experiences of Long Covid patients, including children.

Collaborations between countries are also necessary to further determine Long Covid burden and establish risk factors across geographies and populations, and to estimate impact on health and economic outcomes. Research on strategies that individuals continue to do to prevent and monitor infection and disease, such as masking, testing, use of nasal and throat sprays, other self‐care practices to manage infection and its long‐term sequelae, and use of portable air filters, will continue to provide insights amid relaxed pandemic‐control measures and complacency [13, 27].

Beyond surveillance of COVID‐19 infections, systems need to integrate Long Covid to track burden, recovery, and progression of patients. Although there is progress in identifying affected individuals through the newly introduced 2023 ICD‐10‐CM U09.9 (“Post‐COVID‐19 condition”), more health systems need to adopt this for harmonized reporting and better monitoring. Stronger and more effective public health communication and coordination are also needed: Individuals need to be informed about potential risks even among those who only experience mild symptoms, whereas providers should be up‐to‐date on developments on Long Covid management and treatment.

Finally, Long Covid requires meaningful patient involvement and urgent government policy responses and support, including sick leave, disability benefits, and workplace and school accommodations. Quantifying its prevalence and accelerating the progress on its diagnosis and treatment, particularly in LMICs, will provide information for the design of social insurance benefit packages toward protection from financial risk. This will ensure we meet the needs of those affected by longer term impacts of COVID‐19, recognizing communities and population groups that have been disproportionately affected by it as advocated by Universal Health Care that envisions all individuals will have equitable access to health services.

## AUTHOR CONTRIBUTIONS

*Conceptualization; data curation; investigation; writing—original draft; writing—review and editing*: Arianna Maever Loreche. *Conceptualization; data curation; investigation; writing—review and editing*: Veincent Christian F. Pepito. *Conceptualization; data curation; investigation; supervision; writing—review and editing*: Manuel M. Dayrit.

## CONFLICT OF INTEREST

The authors declare no conflict of interest.
